# Structural Design and Analysis of the RHOA-ARHGEF1 Binding Mode: Challenges and Applications for Protein-Protein Interface Prediction

**DOI:** 10.3389/fmolb.2021.643728

**Published:** 2021-05-24

**Authors:** Ennys Gheyouche, Matthias Bagueneau, Gervaise Loirand, Bernard Offmann, Stéphane Téletchéa

**Affiliations:** ^1^UFIP, Université de Nantes, UMR CNRS 6286, Nantes, France; ^2^Université de Nantes, CHU Nantes, CNRS, Inserm, L'institut Du Thorax, Nantes, France

**Keywords:** PPI, protein-protein docking, molecular dynamics simulation, ARHGEF1, RHOA

## Abstract

The interaction between two proteins may involve local movements, such as small side-chains re-positioning or more global allosteric movements, such as domain rearrangement. We studied how one can build a precise and detailed protein-protein interface using existing protein-protein docking methods, and how it can be possible to enhance the initial structures using molecular dynamics simulations and data-driven human inspection. We present how this strategy was applied to the modeling of RHOA-ARHGEF1 interaction using similar complexes of RHOA bound to other members of the Rho guanine nucleotide exchange factor family for comparative assessment. In parallel, a more crude approach based on structural superimposition and molecular replacement was also assessed. Both models were then successfully refined using molecular dynamics simulations leading to protein structures where the major data from scientific literature could be recovered. We expect that the detailed strategy used in this work will prove useful for other protein-protein interface design. The RHOA-ARHGEF1 interface modeled here will be extremely useful for the design of inhibitors targeting this protein-protein interaction (PPI).

## 1. Introduction

Precise interactions between proteins allow a tight control on many functions and pathways, eventually leading to gene expression or silencing, to protein release or degradation, and even to cell death. To date, there are between 130,000 and up to 650,000 protein-protein interactions (PPIs) described (Ottmann, 2015), but only a fraction of PPIs is validated experimentally, ranging from 14,000 (Rolland et al., 2014) to 125,000 PPIs (http://interactome3d.irbbarcelona.org/, Mosca et al., 2013). This structural gap comes from the difficulties of obtaining experimentally full-length interacting proteins and then to resolving their structures using crystallography, NMR, or electron microscopy (EM). As a result, in most cases for a specific PPI, one has to combine existing incomplete experimental structures with *in silico* approaches. This virtual step is even critical for drug discovery, as examplified with the successful targeting of 50 PPIs by small molecules (Skwarczynska and Ottmann, 2015). When no protein-protein structure is available, one has thus to perform protein-protein docking predictions.

The binding mode prediction of two proteins is very challenging since: (i) minor to major local structural rearrangements may be triggered upon protein recognition, (ii) one protein may recognize multiple proteins, and (iii) cofactors/nucleic acids may be involved to enhance or stabilize the interaction. The analysis of the various existing protein-protein interfaces available in the Protein Data Bank indicates also that (i) the interface area varies greatly between protein families, (ii) the composition in amino acids in this interface may be biased, and (iii) the binding lifetime is transient. Due to the complexity of modeling these diverse PPIs, there are many methods developed, and a global evaluation called CAPRI is performed periodically. We use the most robust and successful methods validated in this competition for protein-protein docking evaluation of the optimal binding mode of our example (Lensink et al., 2007, 2018, 2019).

To illustrate the process of modeling a protein-protein interface, we selected a protein complex where some unbound and bound experimental structures are available, and a complex between other members of the family is available. The first protein partner in our study is RHOA (gene *RHOA*), the second protein partner is Rho Guanine nucleotide Exchange Factor 1 (gene *ARHGEF1*). RHOA is a member of the RAS superfamily of small GTPases recognized as a master regulator of the actin cytoskeleton, thus driving multiple cellular processes, such as cell contraction, migration, proliferation, and gene transcription. While basal and controlled RHOA activity is required for homeostatic functions in physiological conditions, its uncontrolled overactivation plays a causative role in the pathogenesis of several diseases, such as cancer, neurodegenerative, or cardiovascular diseases (Guilluy, 2010; Cherfils and Zeghouf, 2011; Vetter, 2014; Loirand, 2015; Prieto-Dominguez et al., 2019; Arrazola Sastre et al., 2020). RHOA is a molecular switch that couples cell surface receptors to intracellular effector pathways by cycling between a cytosolic inactive state bound to guanosine 5′-diphosphate (GDP), and an active GTP-bound state that translocates to the membrane. The activation of RHOA is mediated by Rho nucleotide guanine exchange factors (GEFs) that promote the exchange of GDP for GTP, which are themselves turned on by the activation of upstream membrane receptors (Cherfils and Zeghouf, 2013). ARHGEF1 is the Rho GEF responsible for the activation of RHOA by angiotensin II through type 1 angiotensin II receptor in vascular smooth muscle cells (Loirand and Pacaud, 2010; Luigia et al., 2015). This signaling pathway participates in the physiological control of the vascular tone and blood pressure, and is causally involved in the pathophysiology of hypertension (Guilluy, 2010).

**Figure 1:**
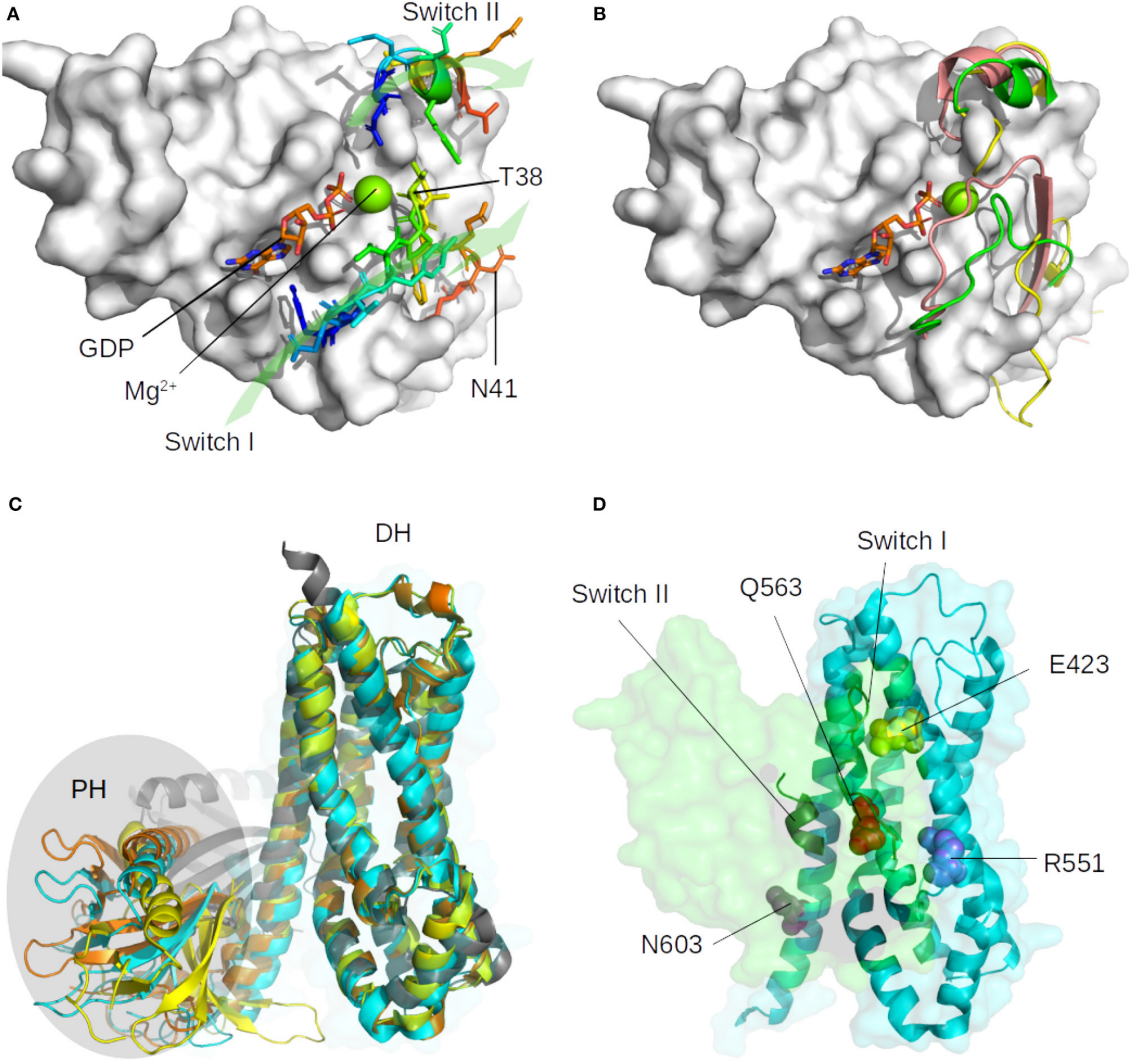
Representation of the major structural changes described for the unbound form of RHOA (1FTN, in white surface), and for the ARHGEF members. **(A)** Location of switch I (loop, 29–42) and switch II (alpha helix, 62–68) with representative residues indicated and green arrows to indicate their orientation. The GDP nucleotide is indicated in orange, blue, and red sticks, the magnesium is shown as a green sphere. **(B)** Diversity of switch I and switch II position as found in representative crystallographic structures. The most mobile Switch I is represented in the tube for the unbound RHOA (green: 1FTN, yellow: 5EZ6, unpublished), and in salmon when bound to the GAP domain of MgcRacGAP (5C2K, unpublished). **(C)** Superimposition on the DH domain of ARHGEF8 (4XH9, yellow), ARHGEF11 (3T06, cyan), ARHGEF12 (1X86, orange), and RHGEF25 (2RGN, gray). The PH domain is highlighted with an oval shape. **(D)** Orientation of RHOA (green surface) on ARHGEF11 (cyan surface) with important conserved residues shown as spheres (see text). Only switch I and switch II on RHOA and DH domain of ARHGEF11 are indicated for clarity.

Small GTPases structure consists of a six-stranded β-sheet (β strands B1 to B6) linked by helices and loops (Ihara et al., 1998). In RHOA, the β-sheet is made up of the anti-parallel association of B1 and B2 and the parallel association of B3, B1, B4, B5, and B6, and there are five α helices (A1, A3, A3′, A4, and A5) and three 3_10_ helices (H1–H3). RHOA possess two hinge regions, a loop called switch I (29–42) and an helix called switch II (62–68), which are described to be more flexible than the core β-sheet (Dvorsky and Ahmadian, 2004), as shown in Figure 1 for various bound and unbound experimental structures. RHOGEF proteins catalyze the exchange of GDP with 5′-triphosphate (GTP) on RHOA (Felline et al., 2019). Two domains on these proteins, Pleckstrin Homology (PH) and Dbl Homology (DH), are involved in the nucleotide exchange mechanism, the RHOA-bound DH domain being more rigid than the PH domain (Figure 1C). The DH domain consists of six α-helices arranged in an oblong shape, which interact with switch I and switch II regions of RHOA. The PH domain contains seven antiparallel β-strands forming a roll architecture, connected to helix α6 of the DH domain. The mechanism of nucleotide exchange involves large displacements of the PH domain relative to the DH-RHOA interaction (Felline et al., 2019).

In this article, we show how to combine virtual approaches with experimental data to predict reliably the formerly non-existing structure of a PPI. By using a rough structural superimposition or more advanced protein-protein docking methods, we build, analyze, and refine the RHOA-ARHGEF1 model and discuss the strengths and weaknesses of this approach. We, then, derive general recommendations to reproduce our approach to model other PPIs.

## 2. Materials and Methods

### 2.1. Sequence Analysis

A BLAST sequence search on the Non-Redundant (NR) database with RHOA or ARHGEF1 sequence as query was performed in June, 2018. The resulting sequences were aligned using clustal Omega (Madeira et al., 2019), amino acids conservation was estimated using Jalview (Waterhouse et al., 2009) and in-house scripts in Biopython (Cock et al., 2009). The phylogenetic analysis of human conserved sequences was performed in Genious version 2019.0.4 (http://www.geneious.com/).

### 2.2. Structure and Interface Analysis

We used all structures of RHOA complexed with all ARHGEFs available in the Protein Databank (Berman et al., 2003) in January 2018 and their respective unbound form when available. Only one representative chain by crystallographic structure was taken as reference (Supplementary Table 1). Experimental structures were analyzed using PDBePISA version 1.54 (Krissinel and Henrick, 2007; Krissinel, 2010). Two methods were selected to analyze protein-protein interfaces in order to obtain useful insights of the important residues involved in the interaction. The first one was 2P2I Inspector, version 2.0 (http://2p2idb.cnrs-mrs.fr/2p2i_inspector.html) (Basse et al., 2016) which computes a series of 51 chemical and physical descriptors from three-dimensional (3D) structures. The second one, PPCheck (http://caps.ncbs.res.in/ppcheck/) is a webserver for quantifying the strength of a protein-protein interface (Sukhwal and Sowdhamini, 2013). It can also be used to predict hotspots, perform computational alanine scanning, and to differentiate possible native-like conformations from the non-native ones given a set of decoy ensembles as obtained through the protein-protein docking as it computes the strength of non-bonded interactions between any two proteins/chains present in the complex. Robetta Server was used to perform virtual alanine scanning of the interface (Kortemme et al., 2004). Models from docking or molecular dynamics simulations were visually assessed and analyzed in The PyMOL Molecular Graphics System, Version 1.8 Schrödinger, LLC. Root Mean Square Deviation (RMSD) was computed using the PyMOL rms_cur command. As RMSD is a global measure, we use two specific measures for rigid body docking and molecular dynamics simulations interface analysis as described in Takemura and Kitao (2019): (i) Ligand-RMSD (L-RMSD) where one protein is Fixed (F) and the second protein is Mobile (M) to compute the L-RMSD. First the Fixed protein is superimposed on the same structure in the crystallographic reference, then the RMSD is computed on the Mobile (M) protein alone. (ii) Interface-RMSD (i-RMSD): in this case, only the amino acids known to be involved in the interface between both proteins are evaluated. Again, a first step consists of superimposing the one protein (RHOA or the PH domain of ARHGEFs) on the reference crystallographic structure to remove translation and rotation degrees of freedom potentially coming from the docking methods process. The angle between helices is computed using a plugin by Thomas Holder, which computes the angle between two vectors created from the coordinates of the Cα atoms of each helix.

### 2.3. Superimposition Model of RHOA-ARHGEF1

A preliminary structure of RHOA bound to ARHGEF1 was derived from the co-crystal of RHOA-ARHGEF11 (PDB id:3T06) (Bielnicki et al., 2011). The complexes were created by superimposition of ARHGEF1 (3ODO) (Chen et al., 2011) on ARHGEF11 in PyMOL. This superimposition was submitted to MolProbity (Williams et al., 2018) for analysis and steric clashes were removed using Chiron (Ramachandran et al., 2011). Chiron performs rapid energy minimization of protein molecules using discrete molecular dynamics with an all-atom representation for each residue in the protein, this process allows to remove most of the steric clashes.

### 2.4. Protein-Protein Docking

The binding mode prediction was done in default mode for all methods using their respective webservers: (i) ATTRACT ff2g (http://chemosimserver.unice.fr/attract/) (Chéron et al., 2017), (ii) ClusPro version 2 (https://cluspro.bu.edu/home.php) (Kozakov et al., 2017), (iii) Haddock (van Zundert et al., 2016), (iv) PyDockWeb, Oct 2017 (https://life.bsc.es/servlet/pydock/home/) (Jiménez-García et al., 2013), (v) ZDOCK version 3.0.2f (Pierce et al., 2014).

### 2.5. Molecular Dynamics Simulation

All-atom simulations of unbound and bound proteins were performed using GROMACS 2016.3 (Abraham et al., 2015), the starting structures are detailed in Supplementary Table 1. Each system was prepared with the AMBER forcefield FF99SB-ILDN (Lindorff-Larsen et al., 2010) in explicit solvent (TIP3P) (Jorgensen et al., 1983) with a specific attention to protonation states as reported in PROPKA. A NVT followed by the anisotropic pressure coupling (NPT ensemble) protocol was applied until equilibration was reached and the full molecular dynamics simulation was computed for 500 ns up to 1μs. All simulations were run on the CCIPL cluster facility at the University of Nantes using GPUs. The force field parameters for GDP and GTP were gathered from the AMBER parameters database (http://research.bmh.manchester.ac.uk/bryce/amber) and converted to GROMACS format files using acpype (da Silva and Vranken, 2012). The resulting trajectories were visualized in the VMD version 1.9.1 (Humphrey et al., 1996), and GROMACS tools were used for various measurements.

PyContact was used to analyze protein contacts type, strength, and lifetime throughout the simulations (Scheurer et al., 2018). These contacts were plot with the R package MDplots (Margreitter and Oostenbrink, 2017).

## 3. Results

In order to determine which docking method was the best for our specific needs, we have evaluated their performance (i) on recovering existing crystallographic structures of RHOA bound to a GEF, a process called re-docking, and (ii) on assembling the bound RHOA from a given crystallographic with a GEF from another crystallographic structure, this process being known as cross-docking.

### 3.1. Protein-Protein Docking Strategies

#### 3.1.1. Z-Dock Is the Best Method for Building Our Complex

As docking strategies are based on different methods, it is difficult to determine *a priori* which method will produce the most reasonable starting complex for further studies. We assessed the top five performing docking software from CAPRI assessment, briefly introduced in Table 1, to produce RHOA/ARHGEF1 complexes: ATTRACT, ClusPro, HADDOCK, PyDockWeb, and ZDOCK.

**Table 1:** Description of the protein-protein docking methods evaluated.

ATTRACT	*Ab-initio* protocol using a coarse-grained forcefield (ff2g) and manage to predict an estimation of the binding energy between the two proteins.	Chéron et al., 2017
CLUSPRO	FFT based program PIPER (pairwise potential based on the decoy at the reference approach) for the docking and using RMSD based clustering for filtering models then scoring using four different Scoring functions	Kozakov et al., 2017
HADDOCK	High Ambiguity-Driven DOCKing allows the use of external data, either experimental or from bioinformatics analysis to drive the modeling process	van Zundert et al., 2016
PyDockWeb	Rigid-body docking using FTDock, Gabb et al. (1997) and Jiménez-García et al. (2013) then an energy scoring based on empirical potential composed by of electrostatic and desolvation terms.	Cheng et al., 2007
ZDOCK	Fast Fourier Transform based protein docking program, version 3.0.2f (IFACE Statistical Potential, Shape Complementarity, and Electrostatics)	Pierce et al., 2014

#### 3.1.2. Assessment of Webserver Performance in Re-docking Experiments

We evaluated the performance of each software according to its ability to recover the existing structure of bound RHOA and ARHGEFs. This method called re-docking allows to discriminate the accuracy of the algorithm studied on our system. The web servers define the first input protein given as the receptor, so it stays fixed (F) and considers the second protein provided as the mobile protein (M). We performed our analysis with the small GTPase or the GEF as (F) or (M). The results are presented in Table 2. We computed the L-RMSD of the predicted complex by superimposing the Fixed protein with the same protein in the crystallographic structure and by computing the RMSD on the Mobile protein to assess the performance of each method. When comparing individually the predicted pose against the reference, ATTRACT and PyDockWeb perform equally with lower L-RMSD values for ATTRACT on its best predictions. ZDOCK and ClusPro present more diverse results and larger deviations. Although it should not be important, we observed that the input order of the fixed and mobile protein was affecting the prediction. This is of limited importance for ATTRACT and PyDockWeb, these methods are, therefore, less sensitive to the size of the mobile protein (RHOA being 200 AA long and GEF DH/PH domains being 600 AA long). We observe that for 4XH9, there is a dramatic decrease in the quality of the prediction although the calculations were performed three times. It is also possible to determine which models ranks the best among all poses and not only the first one: ZDOCK and ATTRACT proposes 10 binding modes, ClusPro offers multiple weights for their scoring functions for clusters of poses, and PyDockWeb presents the 100 best binding poses. All the poses identified as close to the crystallographic structure with a global RMSD under 2 Å were present in the top 10 solutions of the best cluster for each method. Altogether, this lead us to consider the PyDockWeb at the end of the re-docking study, although its computation time is significantly higher than the other methods (Table 2). As ZDOCK and PyDockWeb provided more reliably poses close to the original crystallographic structures, these two methods were kept for the following analysis.

**Table 2:** Analysis of docking software performance for complex formation using small GTPase (RHOA) or GEF (ARHGEF 8/11/12/25) as a mobile (M) or fixed (F) structure.

**GEF(F). RHOA(M)/GEF(M). RHOA(F)**	**ZDOCK**	**ATTRACT**	**ClusPro**	**PyDockWeb**
3T06	1.60/1.92	**0.70/0.97**	3.21/7.10	1.66/1.34
4XH9	1.99/3.06	20.25/14.09^**^	4.27/1.80	**1.52/1.53**
1X86	2.61/3.23	**1.11/1.71**	3.32/5.81	1.44/0.92
2RGN	2.14/3.00	1.46/2.24	3.93/3.01	**0.92/0.80**
Computing time	24 h	24 h	24 h	1 week

*The L-RMSD values are in (Å). The best prediction analyzed is the first of the best 10 poses or the best cluster of poses classified by each method. The best predictions are in bold, the wrong predictions are in italics. HADDOCK could not be evaluated at this stage since we chose to not use data guidance. Computing time is indicative of one docking experiment*.

** *Calculation were performed independently in triplicate to exclude any temporary issue*.

#### 3.1.3. Assessment of the Best Performing Methods in Cross-Docking Experiments

We verified the dependence on the initial structure for the protein-protein binding mode prediction. We applied a cross-docking experiment where each bound RHOA in one crystal structure is evaluated against another GEF partner. We used the free RHOA structure as a sensitivity control for our cross-docking measurements. There is no dependence for the docking result linked to the pdb input for RHOA or GEF. The results are available in Table 3: as one would expect, it is more complicated to build hybrid complexes than re-docking complexes. Out of the 12 combinations of partners for the cross-docking, ZDOCK is better for six predictions, PyDockWeb for five, and their prediction is good and close in one case. For two cases, pydockweb finds a very different orientation than the crystal structure : RHOA: 1X86/GEF: 2RGN (30.27 Å), RHOA: 4XH9/GEF: 1X86 (34.25 Å), where ZDOCK finds a close conformation (3.19/3.31). For both methods, the docking of the unbound RHOA produces very unrealistic protein-protein interfaces, indicating the sensitivity of the method toward switch I and II adaptations of RHOA for GEF binding although the RMSD between bound RHOA (3T06) and unbound RHOA (1FTN) is small (0.7 Å).

**Table 3:** Analysis of the crossdocking performance for the ZDOCK and PyDOCKweb. RHOA was considered as the ligand (M), and each GEF was fixed (F).

**ZDOCK/pydockweb**		**GEF**			
		**GEF11 (3T06)**	**GEF8 (4XH9)**	**GEF12 (1X86)**	**GEF25 (2RGN)**
Bound RHOA	3T06	1.60/1.66	2.15/2.10	3.22/**1.86**	3.51/**2.46**
	4XH9	**3.35**/5.58	1.99/1.52	**3.31**/*34.25*	**2.59**/3.56
	1X86	3.15/**2.46**	2.66/**2.02**	2.61/1.44	**3.19**/*30.27*
	2RGN	**3.01**/5.38	**2.39**/3.38	2.53/**2.16**	2.14/0.92
Free RHOA	1FTN	14.42/29.35	17.02/16.39	31.08/38.60	14.44/38.13

*The RMSD value (in Å) for the mobile part is displayed for the best prediction in the first 10 poses. The results for re-docking experiments, underlined, are identical to Table 2. The best predictions are in bold*.

**Figure 2:**
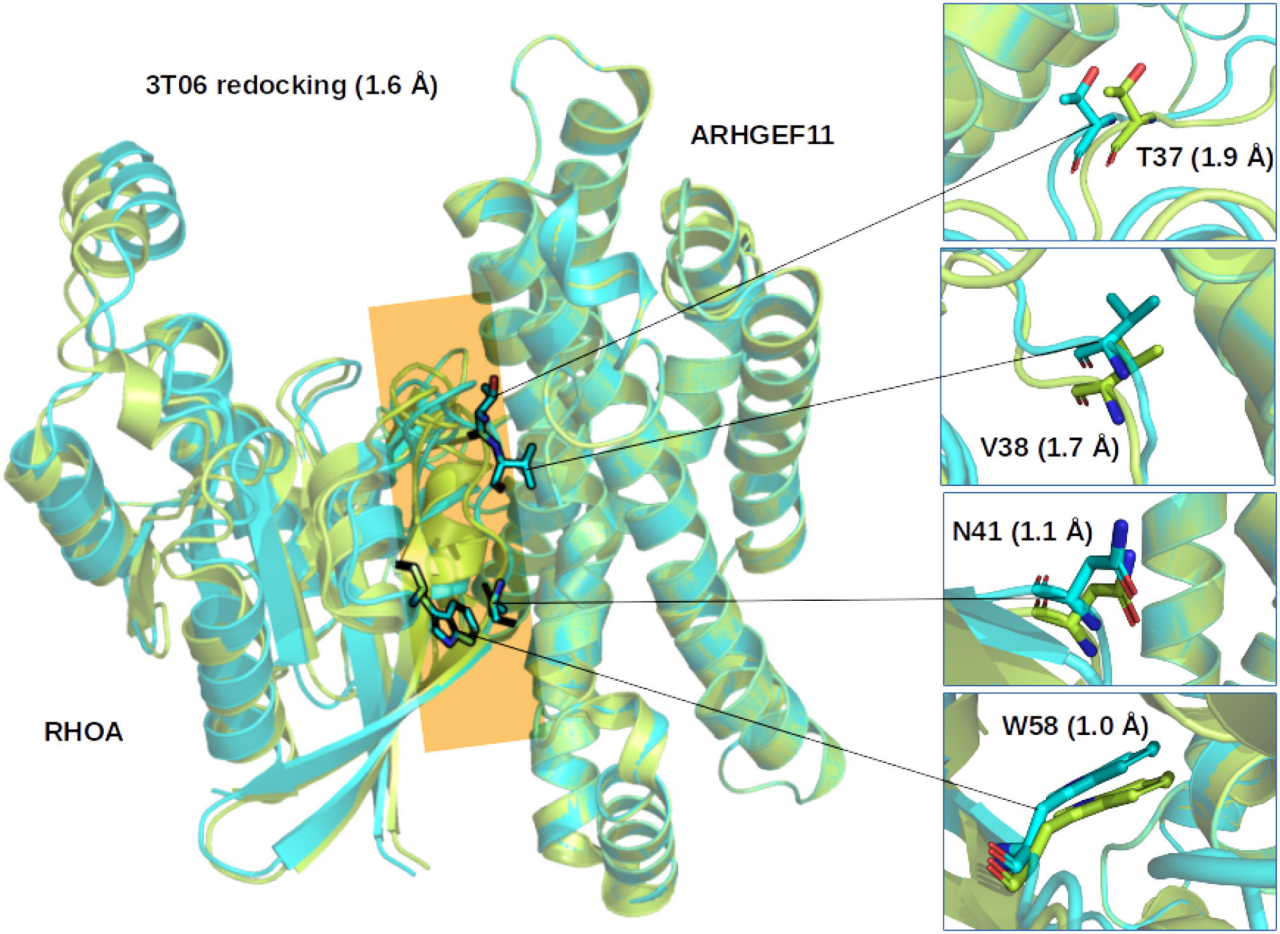
Comparison of the ZDOCK results for 3T06. (Left) Superimposition of the first ranked complex (cyan) on the crystallographic structure (green), the interface between RHOA and ARHGEF11 is indicated by an orange plane. (Right) Zoom on representative RHOA residues determined from multiple sequence alignments to be important for the interface between RHOA and the members of the ARHGEF family. Number in parenthesis indicate the Root Mean Square Deviation on the whole complex or for the selected residues.

Considering the results of the re/cross-docking, we selected ZDOCK as the best method to predict the more favorable binding mode between RHOA and ARHGEF1 (Figure 2).

#### 3.1.4. Evaluation of the Best Models Based on Known Interactions to Select the Best RHOA Candidate Structure

**Figure 3:**
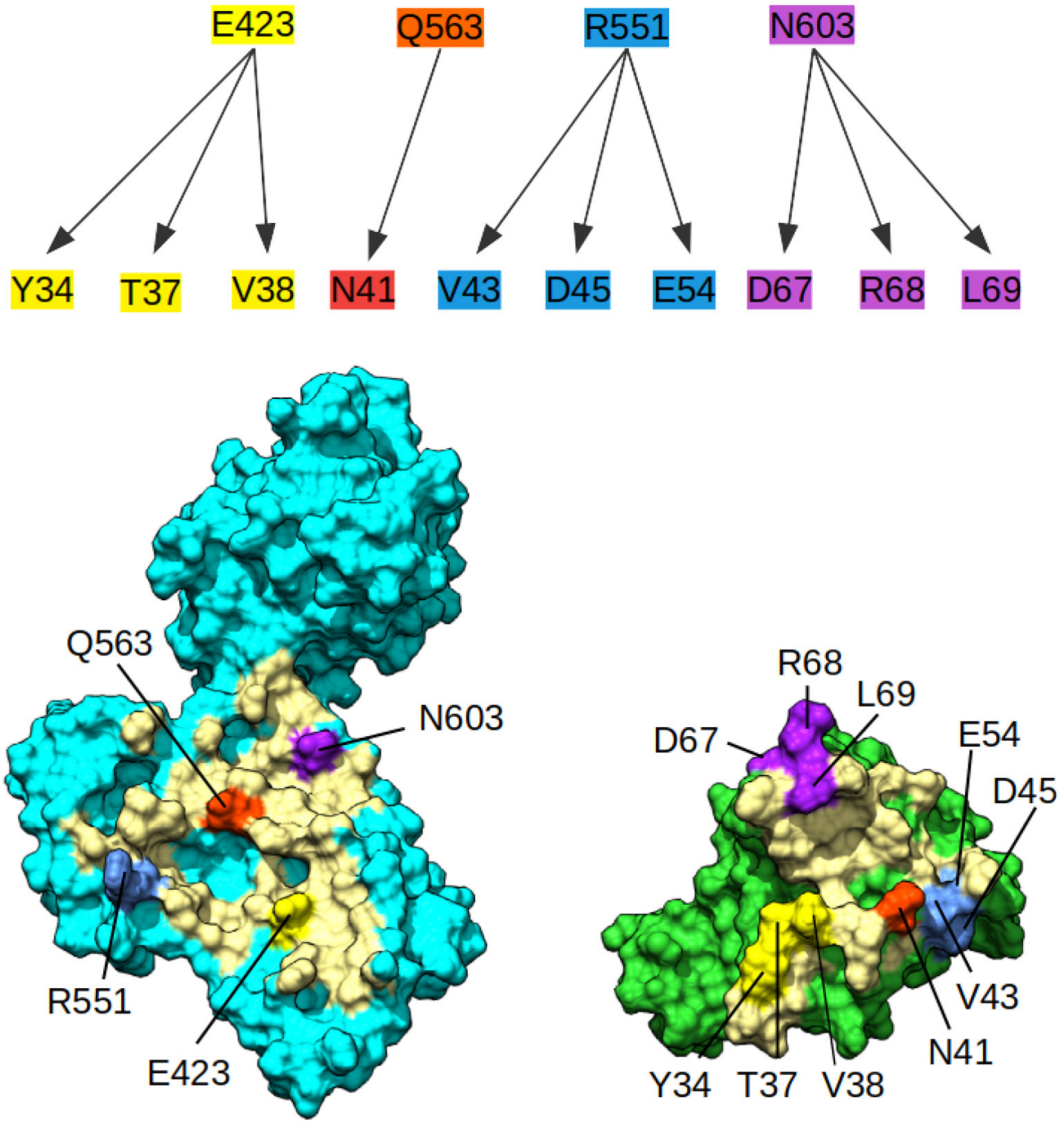
Conserved contacts in the interface between RHOA and all its GEFs. (Top) Diagram of conserved contacts by amino acids, amino acids E423, Q563, R551, and N603 in ARHGEF1, and residues linked by arrows pertaining to RHOA. (Bottom) Split view of ARHGEF1 (cyan) RHOA (green) with matching residues between proteins highlighted in yellow, white, red, blue, and purple, the rest of the interface is indicated in pale yellow.

Since no experimental structure of the RHOA-ARHGEF1 interface is available, we used the crystallographic structures of other GEF paralogs bound to RHOA to determine which amino acids are shared in all complexes. The amino acids mapping to RHOA and ARHGEF1 was done using use multiple sequence alignments comparison (data not shown). Four residues are conserved in GEFs interfaces, namely, E423, Q563, R551, and N603 in ARHGEF1. As can be seen in Figure 3, these shared residues for all GEF interfaces can be split in zones or individual amino acids contacts. By analogy to existing complexes, ARHGEF1 E423 has to be present close to Y34/T37/V38 (a region called switch I in RHOA), R551 has to be close to V43/D45/E54 of RHOA, and N603 has to be close to D67/R68/L69 (a region called switch II in RHOA). Only one amino acid in RHOA, N41 seems to bind exclusively to Q563. It is well-established that RHOA is very rigid due to the strong structural requirements imposed by the GTP recognition and hydrolysis mechanism (Dvorsky and Ahmadian, 2004). Only two regions called switch I and switch II are more flexible with or without binding partners, as seen in Figure 1. Our study allows a more detailed understanding of the interaction between amino acids pairs important for the RHOA-ARHGEF binding.

As done for ARHGEF1, we also analyzed the interface residues of the other GEFs present in each crystallographic structures in the complex with RHOA and assessed their amino acids conservation using multiple sequence alignments. Four residues of ARHGEF1 are present at the interface, and 10 for RHOA. As can be seen in Table 4, we have listed all amino acids present in interaction between both proteins, i.e., at least one amino acid of RHOA is in contact with one amino acid or more of a given ARHGEF. On average, the number of contact pairs recovered after docking represents at least half of the residues known to be present on both sides of the interface. This indicates that our docking strategy allows to build a reasonable starting structure for the RHOA-ARHGEF1 complex. As sequence conservation on the DH+PH domains modeled here is the most important among ARHGEF11, ARHGEF12, and ARHGEF1 (Supplementary Table 2), and provided the docking validation steps showed that ARHGEF11 docking allowed to recover more contacts than with ARHGEF12 docking, we chose the RHOA structure found in 3T06 to dock it using ZDOCK with the unbound structure of ARHGEF1 (3ODO). The resulting complex will be referenced thereafter as complexD (Docking).

**Table 4:** Numbers of amino acids determined to be in interaction between both proteins at the interface, numbers from RHOA (x/10) or its complexed ARHGEF (y/4), as found in the different binding mode generated by ZDOCK during the crossdocking experiments, while the ARHGEF partner was kept fixed.

	**ARHGEF12 (1X86)**	**ARHGEF25 (2RGN)**	**ARHGEF11 (3T06)**	**ARHGEF8 (4XH9)**
RHOA (1X86)	5/10-3/4	5/10-2/4	2/10-1/4	3/10-1/4
RHOA (2RGN)	3/10-3/4	6/10-3/4	8/10-4/4	5/10-2/4
RHOA (3T06)	5/10-1/4	7/10-3/4	6/10-3/4	6/10-2/4
RHOA (4XH9)	3/10-3/4	4/10-2/4	1/10-1/4	7/10-3/4
RHOA (1FTN)	0	0	0	0

### 3.2. Template-Based Complex Modeling

Since there are some experimental structures of RHOA bound to ARHGEF1 homologs, we also predicted the bound structure of both proteins with a simpler approach, based on structural superimposition in PyMOL. We first used a rhoA-bound crystal structure and superimposed the free ARHGEF1 on all the homologous GEFs. By doing so with rigid models, we could not take into account the concerted induced-fit required for finely tuning the interaction. We used a webserver from Dokholyan Team named Chiron which allows to relax the most important steric clashes (http://redshift.med.unc.edu/chiron/) (Ramachandran et al., 2011). In order to solve all the bumps, many rounds were necessary. A preliminary structure of RHOA bound to ARHGEF1 was derived from the RHOA-ARHGEF11 crystal structure (3T06). The complexes were created by superimposition of ARHGEF1 (3ODO) on ARHGEF11 in PyMOL. This superimposition was submitted to MolProbity for analysis, and steric clashes were removed using Chiron. From there, we used classical descriptors to evaluate the resulting complex (delta SAS, RMSD, …) with a special look into the interface size. This interface was analyzed with PDBePISA. The best binding was found when using ARHGEF1 from 3ODO and RHOA from 3T06: the interface is 2,949 Å^2^ corresponding to around 5% of the total surface of ARHGEF1 and to around 11% of RHOA total surface area. In the complexD, this interface comprises amino acids 3 to 181 from RHOA and 392 to 761 from ARHGEF1, for a total interface size of 2,830 Å^2^. Those values are smaller than the values found for the homologs complexes where this interface area is on average 3,371 Å^2^. Before exploring further the complexD and complexT, we verified our models with PPcheck, which decomposes the interaction energy in three terms: (i) hydrogen bonding (Ehyd), (ii) inter-chain van der Waals interactions (Evw), and (iii) inter-chain electrostatic interactions (Eele). The total stabilizing energy is then divided by the total number of interface residues to obtain the energy per residue. No significant deviation requiring further refinements with the Chiron webserver was present in both models.

### 3.3. Molecular Dynamics Simulation Refinement for ComplexT and ComplexD

**Figure 4:**
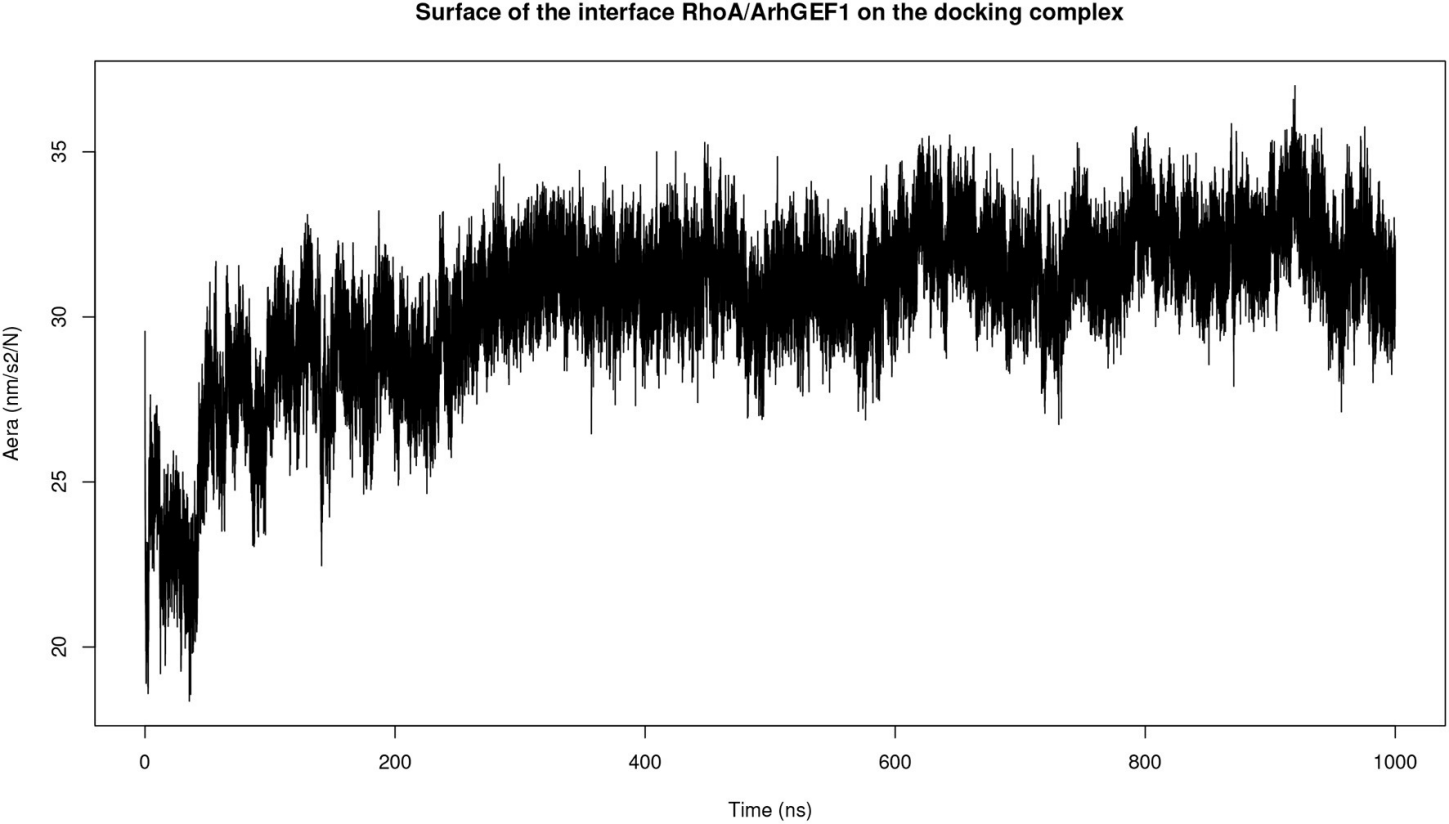
Plot of the interface area between RHOA and ARHGEF1 during molecular dynamics simulation of complexD dock computed using the GROMACS SAS tool.

Both complexes were modeled using molecular dynamics simulations to determine if the interface could be refined during this procedure. The initial surface area in complexD, 2,830 Å^2^ at the beginning of the simulation, stabilizes to 3,056 Å^2^ between 200 and 1,000 ns of the simulation (Figure 4). The molecular dynamics simulation of RHOA-ARHGEF1 complexT also remained stable for most of the time with a rapid initial increase in the interface area followed by a plateau after 250 ns. The average interface size in this plateau is 3,150 Å^2^ (data not shown). Starting with two different models, we observe an augmentation in protein surface contact driven by local adjustments. When considering individually each protein at the RMSD level, there is a higher deviation for RHOA than ARHGEF1, implying that RHOA undergoes most of the conformational changes, as we will see in details below.

### 3.4. Interface Contacts Evolution Over Time

**Figure 5:**
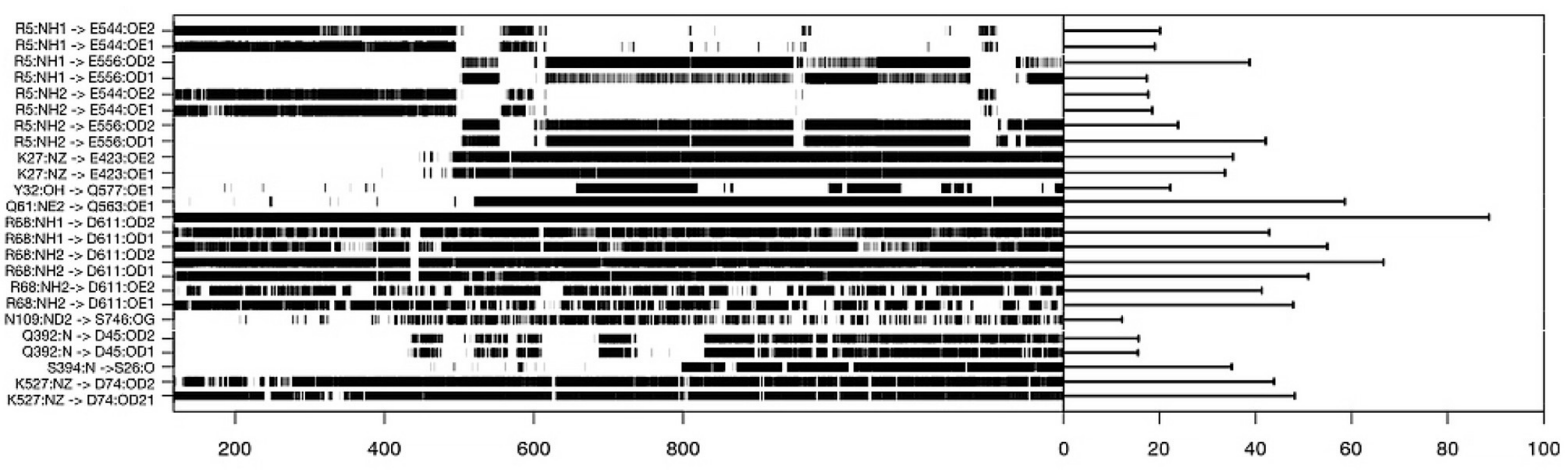
Hydrogen bonds lifetime during molecular dynamics simulation on the complexT, the hydrogen bonds were defined using the hbond routine in GROMACS and analyzed using the MDplot package in R.

To understand the evolution of interface complex during the simulations, we analyzed the hydrogen bonds between the two partners. The result is shown in Figure 5 where we only plotted hydrogen bonds with a lifetime in the simulation over 15%. With this plot, we can identify which amino acids, side chains are seeing important rotations. For instance, an important hydrogen bond is conserved between RHO1-ARG5 and ARHGEF1-GLU544 or ARHGEF1-ASP556. The most stable contact is RHOA-ARG68—ARHGEF1-ASN603/ASP611, since it is observed for 900 ns, or 90% of the simulation time. For others contacts, some were present from the beginning of the simulation, others appeared and disappeared. Since it may take time to stabilize contacts, we observe that an important interaction appears between RHOA-GLN61 and ARHGEF1-GLN563 at 500 ns. Interestingly, some of these hydrogen bonds are members of the very conserved list of amino acids listed above, for instance, for RHOA-ARG68 and ARHGEF1-ASN603.

## 4. Discussion

Since no experimental structure of RHOA-ARHGEF1 was available from X-ray studies, NMR, or EM studies, we had to model it. Using the protein sequences of Rho and GEF families, and the existing protein structures of bound homologs RHOA-ARHGEF8 (Petit et al., 2018), RHOA-ARHGEF11 (Bielnicki et al., 2011), RHOA-ARHGEF12 (Kristelly et al., 2004), and RHOA-ARHGEF25 (Lutz et al., 2007), we analyzed to determine which amino acids were shared at the interface of the complex (Figure 3). This sequence and structure-based information was important to assess the validity of our models.

### 4.1. Initial Models of RHOA-ARHGEF1 Complex

The prediction of protein-protein interface using docking methods is still an important field of research (Smith and Sternberg, 2002; Lensink et al., 2007) but the predictive power of these methods greatly varies depending on the protein families (Bendell et al., 2014; Wang et al., 2017). As no GEF or RHOA experimental structure was used as target to assess the methods in recent CASP experiments, we benchmarked how these methods could perform on our specific case, using re-docking and cross-docking experiments. We selected ZDOCK after a careful quantification and inspection of the re-docking/cross-docking experiments since its results were the most robust across most predictions and in agreement with our sequence+structure derived data. The best model (**complexD**) selected from ZDOCK contains 5 out of 10 shared amino acids in RHOA and 3 out of 4 shared amino acids from ARHGEF1, with an interface surface area of 2,830 Å^2^.

As the docking experiments are time-consuming and contain also uncertainties, we did also a more crude approach using PyMOL. We analyzed the existing structures of RHOA bound to other members of the ARHGEF family to find the best starting template for our structural comparison. A superimposition of ARHGEF1 (3ODO) on RHOA-ARHGEF11 (3T06) was then performed in PyMOL and further refined using Chiron (Ramachandran et al., 2011). This modeled interface (**complexT**) allowed to correctly find the position of 2 out of 10 amino acids in RHOA and 2 out of 4 amino acids from ARHGEF1.

Both methods allowed to define comparable starting complexes of the RHOA-ARHGEF1 interface from rigid templates. Major steric clashes were carefully examined using Chiron (Ramachandran et al., 2011) and visual inspection, but no further amino acids adjustment was required. The RMSD difference between both models is 0.4 Å for ARHGEF1 and 9.5 Å for RHOA. This larger difference in RHOA position comes from an alternate orientation of the protein relative to ARHGEF1 with a clockwise rotation of 22° between RHOA in complexT and RHOA in complexD (**Figure 7**). This alternative positioning of RHOA in comparison to other members of the GEF family is also present in crystallographic structures. Both complexT and complexD seemed therefore reasonable starting complexes, with a comparable building time of 1 day for both protocols: instant for PyMOL superimposition plus 1 day for removal of clashes in Chiron and 1 day for ZDOCK prediction.

### 4.2. Molecular Dynamics Interface Refinement

**Figure 6:**
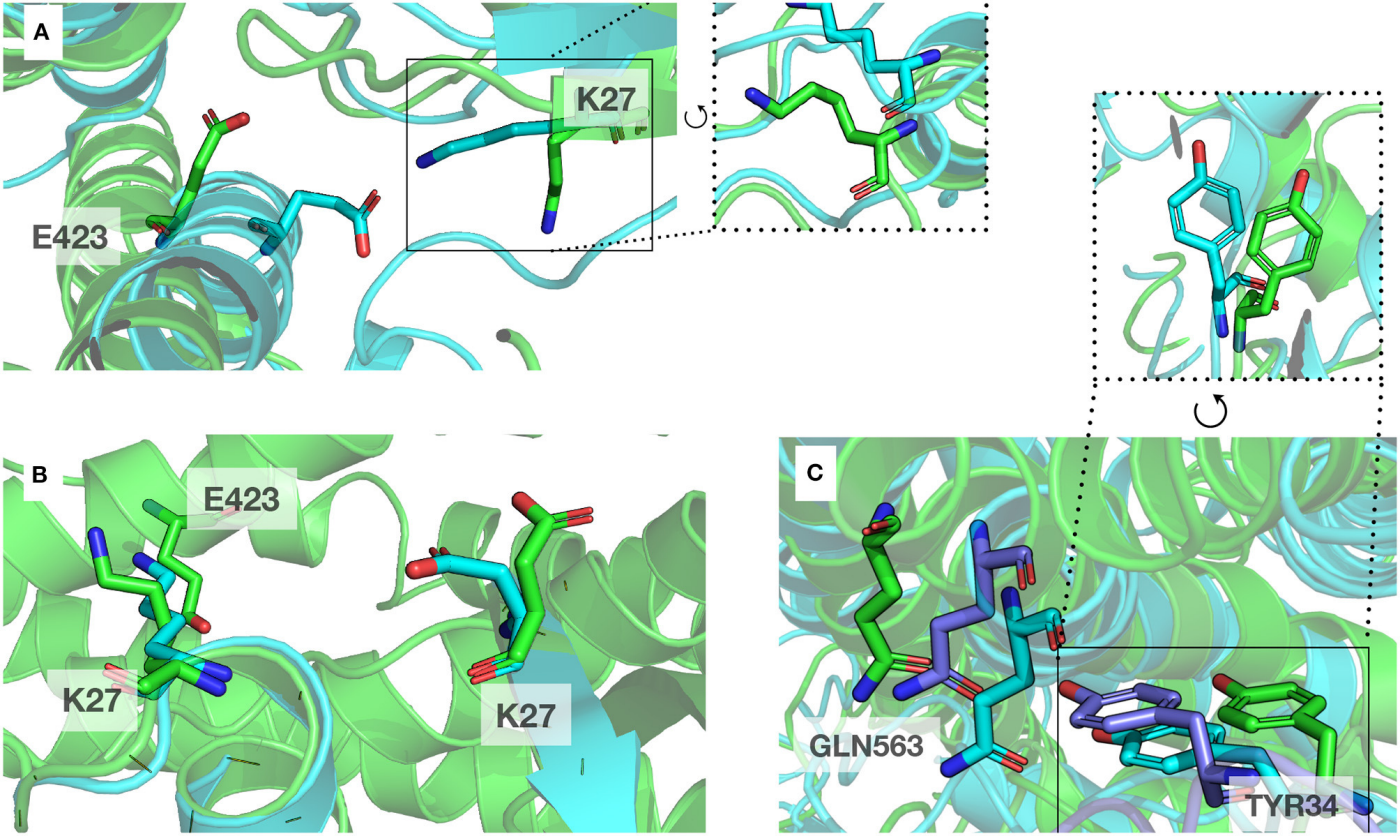
Example of local rearrangements observed at the interface of both proteins. The initial position displayed in cartoon and sticks representation are in green, and the optimized positions are in blue. **(A,B)** Reorientation of E423 (ARHGEF1) and K27 (RHOA) seen from different orientations, **(C)** Green, blue, and purple: representative discrete positions of GNL563 (ARHGEF1) and TYR34 (RHOA).

A classical method to enhance protein models is molecular dynamics simulations (MD) (Mirjalili et al., 2014). We performed MD on complexT and complexD for 1μs each. During this simulation of the complexT, the interface area in the complex increased (3,480 Å^2^) in comparison to the initial complex (2,949 Å^2^). We identified amino acids conserved in all RHOA-ARHGEF complexes by combining structural sequence analysis (Figure 3A). These contacts are stable throughout the simulation (R5, R68/E544, D556, N603, and D611). Interestingly new contacts are observed K27-E423, R68-D611 led by local rearrangements of amino acids, in particular K27, Y34 (RHOA), and E423 (ARHGEF1) (Figure 6).

Both complexT and complexD lead to a similar RHOA-ARHGEF1 interface at the end of the simulation. At the beginning of the simulation (*t* = 0 ns), ComplexD and complexT only have a global RMSD difference of 1.4 Å between them, but at the end (*t* = 1,000 ns), the RMSD rises to 3.7 Å. Since the RMSD is a global measure of the movements, i.e., both proteins moved, it is important to understand how proteins evolved independently during the simulations.

**Figure 7:**
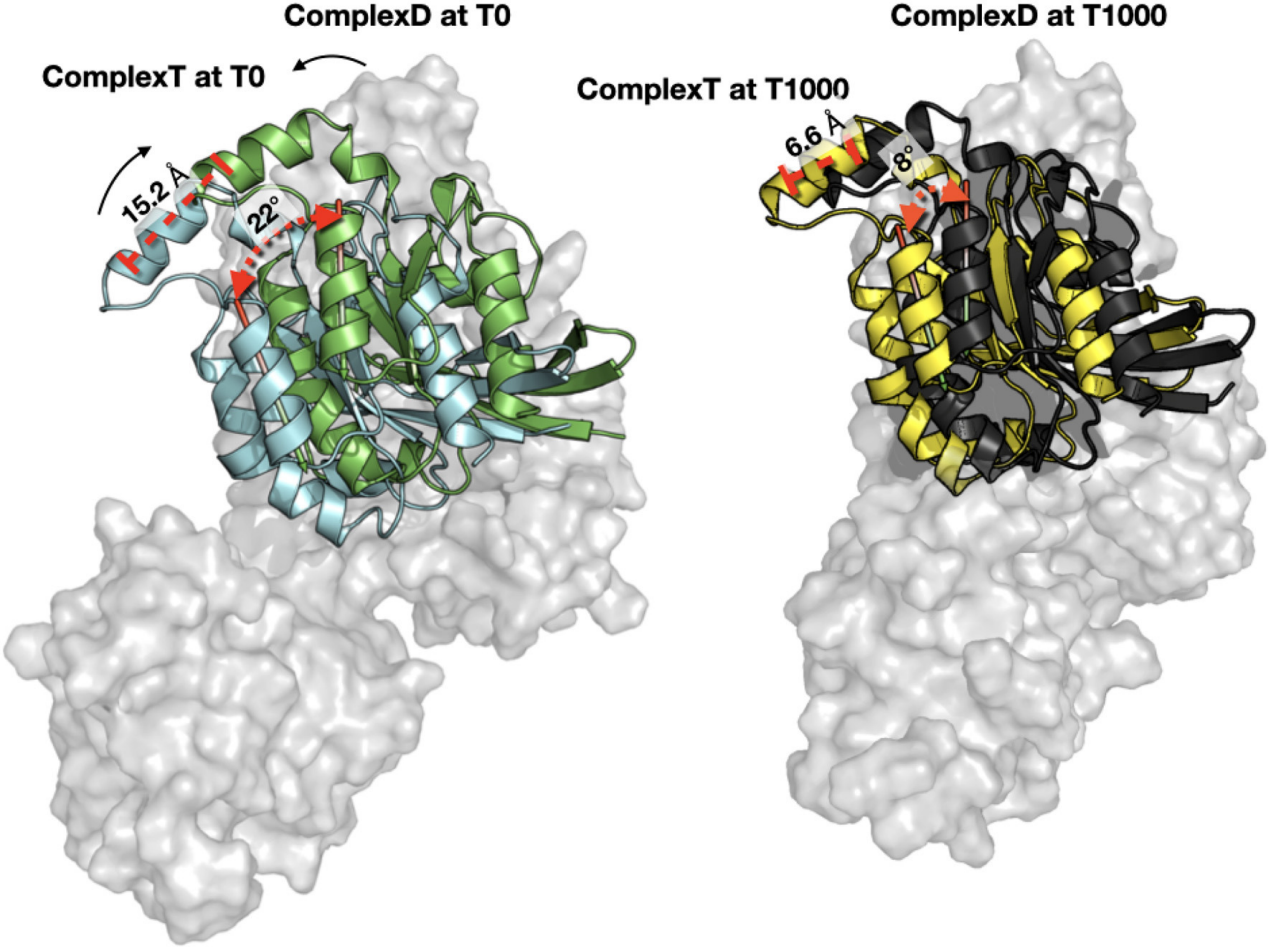
Orientation of RHOA relative to ARHGEF1. (Left) Comparison of RHOA in complexT (blue) and RHOA in complexD (green) at the beginning of the simulation (T0). Only the surface of ARHGEF1 (gray) of complexT is shown for clarity. The center of one helix of RHOA is displayed in red stick to illustrate the clockwise movement observed during the simulation, with an angle of 22.47° and a distance of 15.2Å between the top of the helix. (Right) Same orientation with the same angle and distance between the last snapshot of complexT (yellow) and complexD (black), the shift in both complexes is only 8.66° for a distance of 6.6Å. ARHGEF1 of complexT is displayed in transparent gray surface, since ARHGEF1 proteins are aligned on the PH domain, the difference in the bottom of the figure comes from the movement of the DH domain.

When each trajectory is taken individually, we observe that ComplexD moved more (5.1 Å) from its initial structure than complexT (2.9 Å). This apparent difference comes mostly from a larger movement of the PH domain in the complexD simulation since the RMSD between the initial structure and the end of the simulation concerning only the ARHGEF1 protein is 18 Å. This apparently large difference in ARHGEF1 position for complexD is the consequence of two movements: (i) the local rearrangements of the DH domain (the core RHOA binding domain of GEFs), which is similar in both complexD and complexT (6 Å), and (ii) a larger movement of the PH domain which may be involved in the nucleotide exchange. When aligning the DH domain in both trajectories, we, therefore, see a more important rotation of RHOA relative to ARHGEF1 for complexD (6 Å) than for complexT (3.5 Å) as illustrated in Figure 7. The main interface enhancements thus appear locally at the ARHGEF1-RHOA interface, mostly on the DH domain of ARHGEF1, and more globally with a clockwise (+22°) rotation in the complexT, and a slighter anti-clockwise (−3°) rotation in complexD.

Both complexes are refined after molecular dynamics simulations, many important amino acids saw an increase in contact frequency and the position of RHOA relative to ARHGEF1, either inherited from the superposition onto ARHGEF11 or from the docking studies with ZDOCK, led to a strong convergence of the interaction. This study confirms the interest of using molecular dynamics simulation to increase model quality.

### 4.3. Validation of the Binding Mode

**Figure 8:**
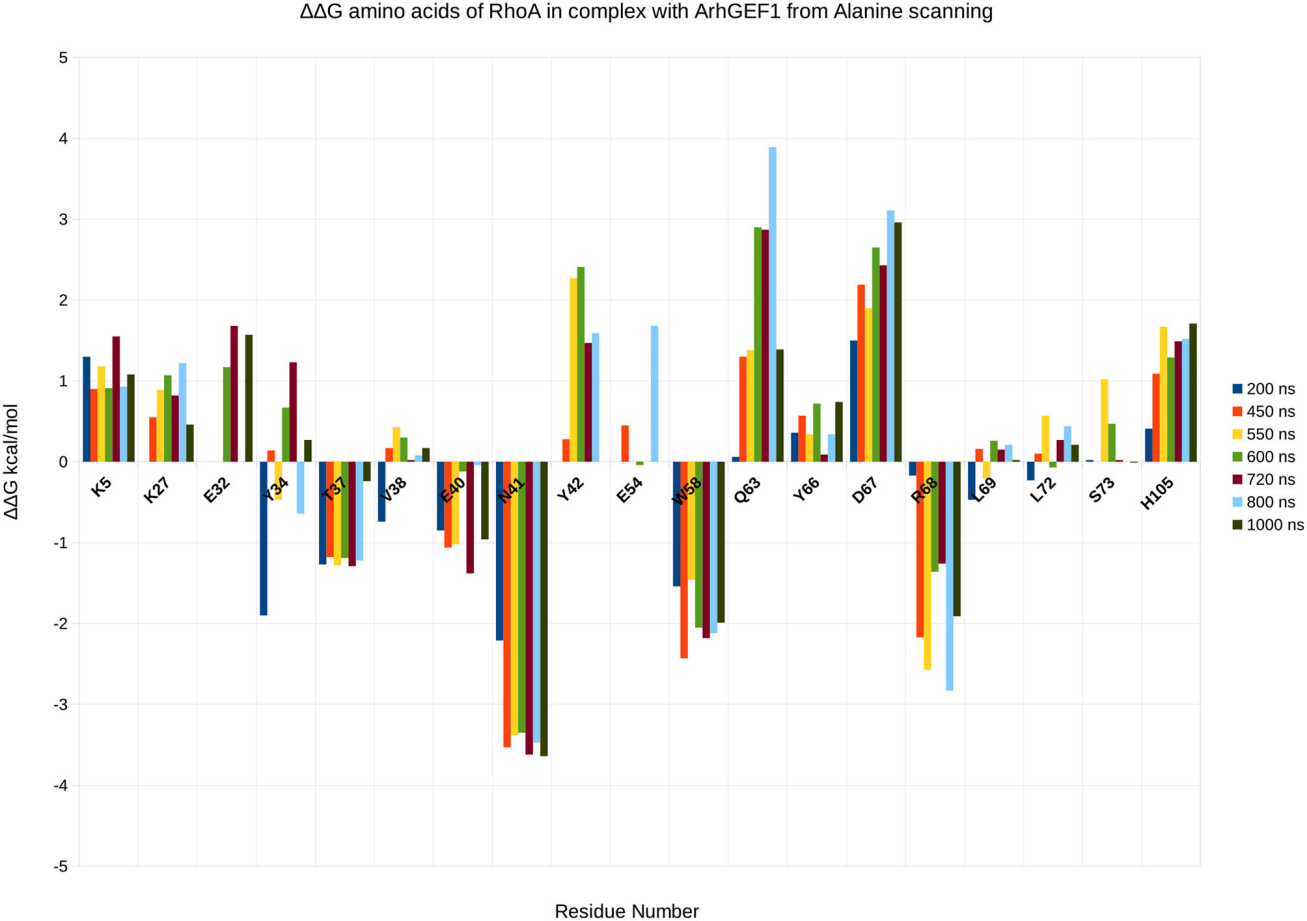
Energy contribution of RHOA amino acids present at the interface for different snapshots of the simulation, when important shifts in hydrogen bond networks were observed. The displacement is computed as a virtual alanine scanning using the Robetta webserver.

**Figure 9:**
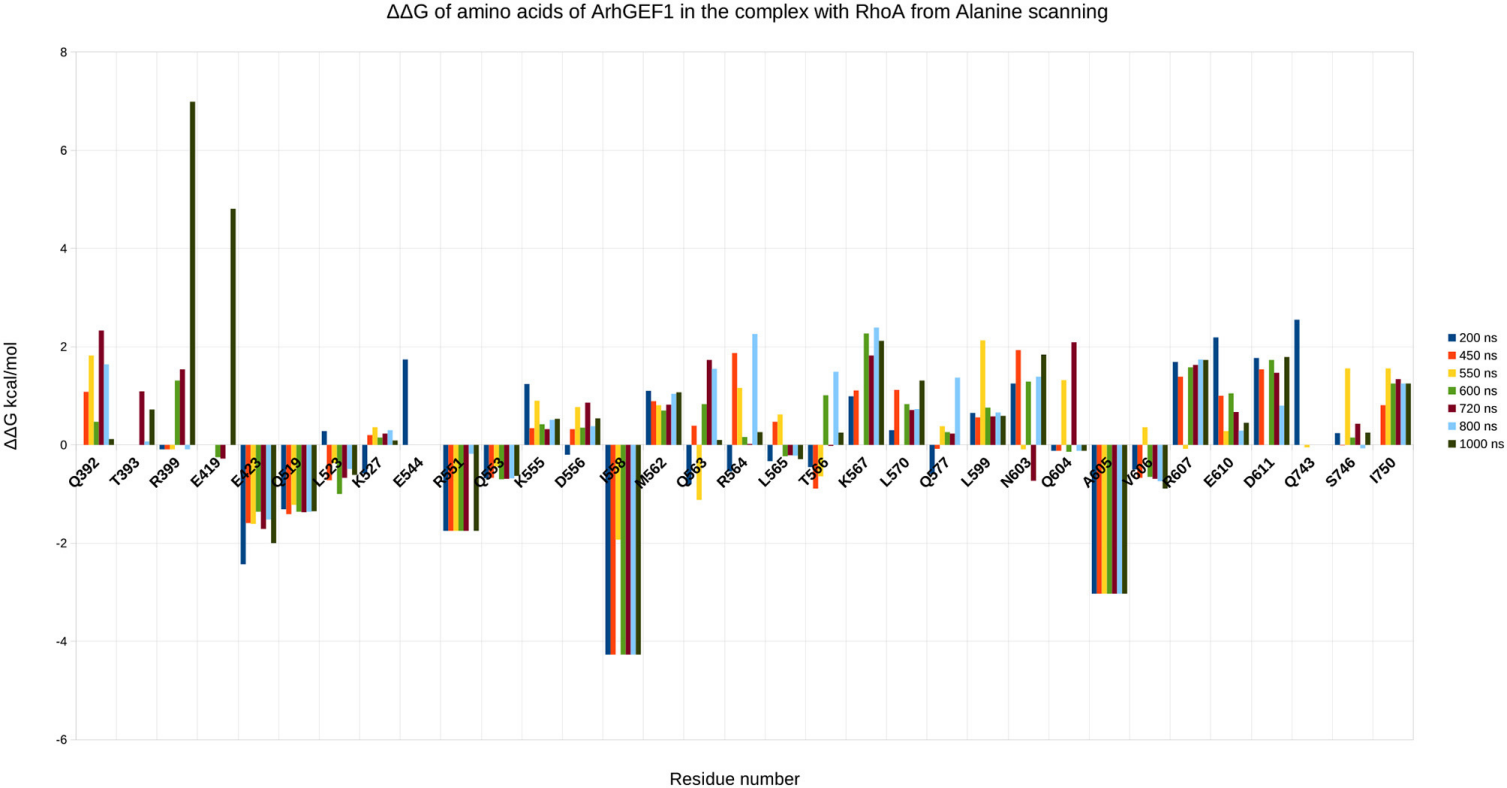
Energy contribution of ARHGEF1 amino acids present at the interface for different snapshots of the simulation, when important shifts in hydrogen bond networks were observed. The displacement is computed as a virtual alanine scanning using the Robetta webserver.

To validate the binding mode from the simulations, we used the interactions as a starting point and analyzed specific interaction of this complex found in the literature. We found back couples of interactions, which have a lifetime of over 15%, are conserved in all RHOA/GEFs binding modes, with some interactions specific to the RHOA-ARHGEF1 interface. We observed that the complex tends to go toward a more stable conformation when the PH domain moves to enclose RHOA, with an increase in the number of interactions, with a mean SAS going over 3,100 Å^2^, the mean surface area for all complexes of RHOA/GEFs available so far. We could identify some specific contacts for RHOA-ARHGEF1 from the complexT, namely D59-K567, Q63-T566, which were not described previously (Hoffman and Cerione, 2002; Derewenda et al., 2004). In the complexD, the specific contacts E97-S746 has already been described by Gasmi-seabrook (Gasmi-Seabrook et al., 2010) as an essential contact in the nucleotide exchange for PDZ-ARHGEF1 and RHOA. This contact is not observed in the complexT simulation. Starting from two complexes built with different strategies, we were able to have a perfect compatibility between experimental predictions and our *in silico* methods. The identified additional contacts, specific for the RHOA-ARHGEF1, will require experimental exploration since there were differences from the two simulations, potentially coming for the overall dynamics of the interface. To guide experimental validations, we studied *via* virtual alanine scanning if some amino acids could be qualified as hotspots (Kortemme et al., 2004; Jiang et al., 2017) of the interface (Figures 8, 9). Only three amino acids seem to contribute strongly to the binding of both proteins: (1) N41 for RHOA, already identified by multiple sequence alignments and structure comparison, (2,3) I558 and A605 in ARHGEF1. These residues seems to be robustly involved in the interface during all the simulations, either starting with the complexD or the complexT.

### 4.4. Selection of the Best Model

The knowledge acquired with our strategies helped us to understand the most relevant elements for the binding of the two partners altogether with insights for selecting/computing relatively good refined models. In the initial models after minimization for complexT, there were already 5 over the 10 conserved (E423, Q563, and N603) contacts and for the complexD 4 over 10 (mostly with E423). After simulation for complexT, we can see 8 over 10 conserved contacts and for complexD, there are 6 over 10 contacts during a short time frame where the interface SAS is the highest. Some contacts are seen only thanks to the simulations and one question rises, what is more important to select for qualifying the best model? Its higher number of contacts or the presence of conserved/important contacts? In our case, both models show conserved/important contacts and new contacts specific to each model. The amino acids detected as hotspots are not conclusive since they are present in both trajectories. During the simulations, even if starting from somewhat different structures, the interaction between ARHGEF1 and RHOA converges. Our model building strategy clearly indicates that a molecular dynamics simulation, starting from rationally designed PPI, improves the initial models.

### 4.5. Comparison of the MD Model With Information-Driven HADDOCK Docking

**Figure 10:**
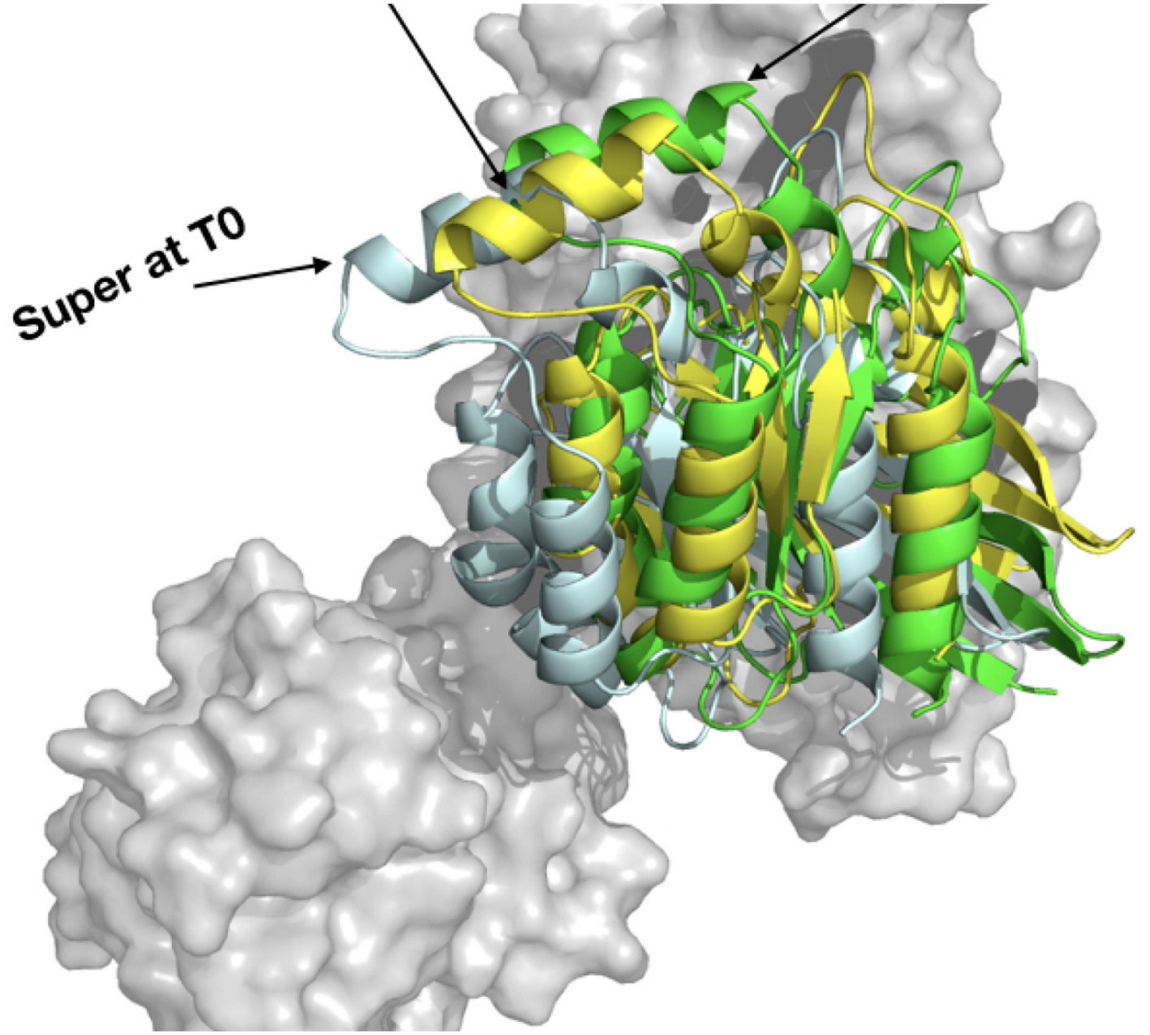
Comparison of the HADDOCK webserver cluster 1 model built from 3T06 RHOA and 3ODO ARHGEF1 (yellow), complexT (light cyan), and complexD (green).

HADDOCK is very efficient for protein-protein docking when experimental/bioinformatics constraints can be added for driving the docking. As we had determined the important residues for the binding interface, we used them in HADDOCK webserver first for redocking experiments on the 3T06 crystal structure as shown for other methods in Table 2, and obtained a RMSD of 0.75 Å with 90% of the structure in the first same cluster, better than all other protein-protein docking methods. We then did the prediction of the interaction model using ARHGEF1 from 3ODO and RHOA from 3T06. This prediction gave us nine clusters, and after careful analysis only two seemed to have the expected binding mode: cluster 1 and cluster 5. When cluster 1 is compared to ZDOCK's derived complexD (without information driven construction), this cluster 1 has a RMSD of 0.62 Å, also very close to complexT with a RMSD value of 0.65 Å (Figure 10). Qualitatively, the cluster1 model displays 6 over 10 of the contacts given as input. If experimental data are available, for instance, coming from mutagenesis experiments, HADDOCK allows their incorporation to guide the binding mode. In this situation, HADDOCK is certainly the best strategy to build a PPI, provided these data can be transformed in sufficient constraints as input. However, the resulting model provided by HADDOCK in 1 day compared to other docking methods is very interesting. The interface area for cluster 1 is 1,478 Å^2^, slightly better than complexD model before refinement (1,420 Å^2^), but far from the refined interface obtained after molecular dynamics simulations (>3,000 Å^2^). Even if it is possible to build a reliable model by integrating various data in HADDOCK, a long molecular dynamics simulation, with a simulation time above 250 ns is still required to enhance the quality of a PPI (Feig and Mirjalili, 2016).

## 5. Conclusion

Most biological processes involve transient protein-protein interactions, in particular for cellular signaling. The RHOA-ARHGEF1 interaction is responsible for the activation of RHOA downstream of type 1 angiotensin II receptor signaling in vascular smooth muscle cells, thereby controlling vascular tone and blood pressure (Loirand, 2015).

**Figure 11:**
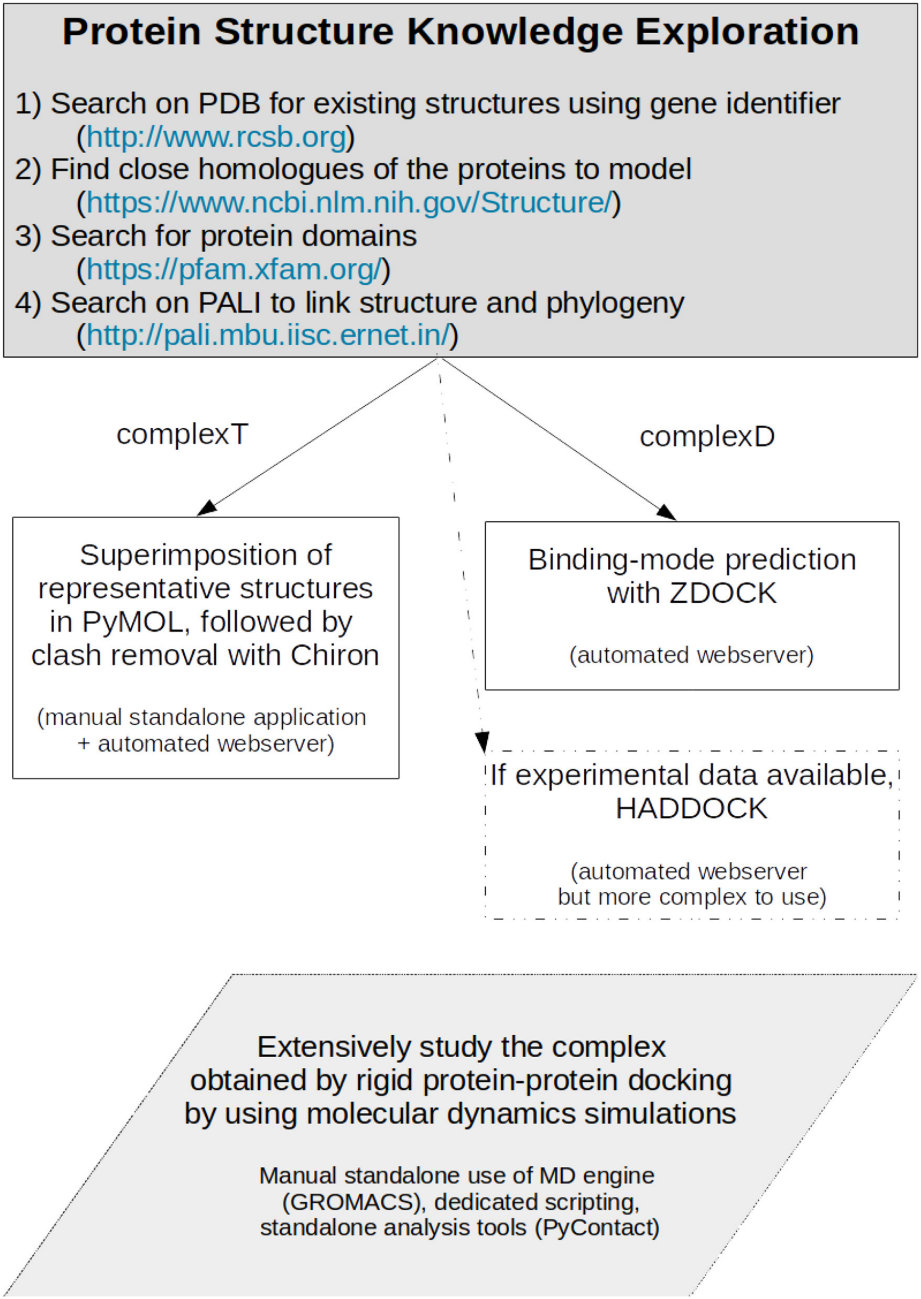
Protocol for producing a protein-protein complex where close homologs of the proteins of interest can be identified.

Our study aims at exemplifying how one can model a protein-protein interaction when sufficient experimental structures are present, but only experimental data for close homologs are available. We set up two different strategies summarized in Figure 11. One has first to identify the close homologs. If the members of the family have a standardized name, they should rapidly be identified directly in the Protein Data Bank (Berman et al., 2003). If not, a search on the National Center for Biotechnology Information (NCBI) structure service, in the Protein Families Database (Mistry et al., 2021), or in the PALI database (Balaji et al., 2001) should help in finding close homologs. If not, the protocols described in our work should be considered with caution. When the close homologs are identified, it is possible to apply the protocols previously described. The first one, based on structural superimposition of partners, allows a rapid building of the complex, but provides required local adjustments to avoid steric clashes. We expect it to be useful for a preliminary study of how the proteins interact. The second strategy based on most advanced methods combining the search of the best binding mode *via* the assessment of the results of protein-protein docking, followed by the refinement of the best docked model using molecular dynamics simulations. This model showed not only increased shape complementarity and increased contacts but also provides insights into the dynamics of the detailed amino acids interactions between the partners. This more advanced strategy is probably only accessible to experts and should only be required for atomic-level analysis and mechanistic studies. In our study, both strategies gave close initial models, but we do not expect the results on RHOA-ARHGEF1 to be amenable for general purpose. We, therefore, recommend to use a protein-protein rigid-body docking study (complexD) for producing the initial interaction mode. In our study, ZDOCK was better if precision, robustness, and time are taken altogether into consideration. When possible, we recommend to perform long molecular dynamics simulations to enhance the network of interaction between both proteins and to get a better overview of the lifetime of each interaction. More generally, we expect these strategies will be successfully applied to a variety of targets where a partial structural coverage of both partners is known, provided the complex to model has characteristics comparable with the two proteins described in this article.

## Data Availability Statement

The datasets presented in this study can be found in online repositories. The names of the repository/repositories and accession number(s) can be found in the article/Supplementary Material.

## Author Contributions

ST and GL obtained the funding. EG did the experiments and analysis. BO and GL supervised the work. EG and ST wrote the manuscript. All authors contributed to the article and approved the submitted version.

## Conflict of Interest

The authors declare that the research was conducted in the absence of any commercial or financial relationships that could be construed as a potential conflict of interest.

## Abbreviations

RMSD: root mean square deviation
SAS: solvent accessible surface.
