# Supplementary material for: Structural Design and Analysis of the RHOA-ARHGEF1 Binding Mode: Challenges and Applications for Protein-Protein Interface Prediction
Source: Front Mol Biosci. 2021 May 24;8:643728. doi: 10.3389/fmolb.2021.643728 (PMC8181724; doi:10.3389/fmolb.2021.643728)
Supplement: Supplementary file 1 [file Data_Sheet_1.PDF]

Supplementary Material

1 FIGURES

2 TABLES

Table S1. Protein structure identifiers of free and bound RHOA and ARHGEF proteins.

Id	Title	ARHGEF	Interface (Å^2)	Reference
1X86	Crystal Structure of the DH/PH domains of Leukemia-associated RhoGEF in complex with RhoA	12	1528	Kristelly et al. (2004)
2RGN	Crystal Structure of p63RhoGEF complex with Galpha-q and RhoA	25	1386	Lutz et al. (2007)
3T06	Crystal Structure of the DH/PH fragment of PDZRHOGEF with N-terminal regulatory elements in complex with Human RhoA	11	1572	Bielnicki et al. (2011)
4XH9	Crystal Structure of Human RHOA in Complex With DH/PH Fragment of the Guanine Nucleotide Exchange Factor NET1	8	1840	Petit et al. (2018)
1FTN	Crystal Structure of the Human RHOA/GDP Complex		free RHOA	Wei et al. (1997)
3ODO	Crystal Structure of the DH/PH Domains of p115-RhoGEF	1	free ARHGEF1	Chen et al. (2011)

Table S2. Protein sequence conservation of ARHGEF proteins with a bound structure to RHOA. Only the PH and DH domains are considered for sequence identity conservation calculation. Domain definitions are taken from PROSITE annotations (Sigrist et al. (2013)) as found in UniProt (The UniProt Consortium (2021)). Sequence conservation was computed as a global alignment with DH+PH domains of ARHGEF1 using the needle command of the EMBOSS suite (Rice et al. (2000))

ARHGEF	UniProt Id	PH+DH position	DH+PH size (AA)	%id	% similarity
1	Q92888	416-760	344	100.0	100.0
8	Q7Z628	174-501	327	25.1	42.6
11	O15085	345	734-1079	**52.7**	**73.8**
12	Q9NZN5	345	787-1132	51.7	73.3
25	Q86VW2	306	160-466	18.8	31.8

REFERENCES

Kristelly R, Gao G, Tesmer JJG. Structural Determinants of RhoA Binding and Nucleotide Exchange in Leukemia-associated Rho Guanine-Nucleotide Exchange Factor. *Journal of Biological Chemistry* **279** (2004) 47352–47362. doi:10.1074/jbc.M406056200.

Lutz S, Shankaranarayanan A, Coco C, Ridilla M, Nance MR, Vettel C, et al. Structure of gαq-p63rhogef-rhoa complex reveals a pathway for the activation of rhoa by gpcrs. *Science* **318** (2007) 1923–1927. doi:10.1126/science.1147554.

Bielnicki JA, Shkumatov AV, Derewenda U, Somlyo AV, Svergun DI, Derewenda ZS. Insights into the Molecular Activation Mechanism of the RhoA-specific Guanine Nucleotide Exchange Factor, PDZRhoGEF. *Journal of Biological Chemistry* **286** (2011) 35163–35175. doi:10.1074/jbc.M111.270918.

Petit AP, Garcia-Petit C, Bueren-Calabuig JA, Vuillard LM, Ferry G, Boutin JA. A structural study of the complex between neuroepithelial cell transforming gene 1 (Net1) and RhoA reveals a potential anticancer drug hot spot. *Journal of Biological Chemistry* **293** (2018) 9064–9077. doi:10.1074/jbc.RA117.001123.

Wei Y, Zhang Y, Derewenda U, Liu X, Minor W, Nakamoto RK, et al. Crystal structure of RhoA-GDP and its functional implications. *Nature Structural Biology* **4** (1997) 699–703.

Chen Z, Guo L, Sprang SR, Sternweis PC. Modulation of a GEF switch: Autoinhibition of the intrinsic guanine nucleotide exchange activity of p115-RhoGEF. *PROTEIN SCIENCE* **20** (2011) 107–117. doi:10.1002/pro.542.

Sigrist CJA, de Castro E, Cerutti L, Cuche BA, Hulo N, Bridge A, et al. New and continuing developments at PROSITE. *Nucleic Acids Research* **41** (2013) D344–347. doi:10.1093/nar/gks1067.

The UniProt Consortium. UniProt: the universal protein knowledgebase in 2021. *Nucleic Acids Research* **49** (2021) D480–D489. doi:10.1093/nar/gkaa1100.

Rice P, Longden I, Bleasby A. EMBOSS: The European Molecular Biology Open Software Suite. *Trends in Genetics* **16** (2000) 276–277. doi:10.1016/S0168-9525(00)02024-2.
